# Supplementary material for: Moderated estimation of fold change and dispersion for RNA-seq data with DESeq2
Source: Genome Biol. 2014 Dec 5;15(12):550. doi: 10.1186/s13059-014-0550-8 (PMC4302049; doi:10.1186/s13059-014-0550-8)
Supplement: Additional file 1 — Supplementary methods, tables and figures. [file 13059_2014_550_MOESM1_ESM.pdf]

Supplementary Materials for:

Moderated estimation of fold change and dispersion for RNA-seq data with DESeq2

Michael I Love [1,2,3], Wolfgang Huber [1], Simon Anders [1,*]

[1] Genome Biology Unit, European Molecular Biology Laboratory, Heidelberg, Germany
[2] Department of Biostatistics and Computational Biology, Dana Farber Cancer Institute and Department of Biostatistics, Harvard School of Public Health, Boston, MA, USA
[3] Department of Computational Molecular Biology, Max Planck Institute for Molecular Genetics, Berlin, Germany
[*] Corresponding author (email: sanders@fs.tum.de)

Supplementary Methods

Benchmarking code

The code used to run the count-based algorithms is contained in the file `inst/script/runScripts.R` in the *DESeq2paper* package (available at http://www.huber.embl.de/DESeq2paper). The code for the simulations is referenced from the simulations vignette in this package. The code which ran the algorithms over the real datasets is contained in the files `inst/script/pickrell/diffExpr.R` (the specificity analysis run on the Pickrell et al. [1] dataset) and `inst/script/bottomly/diffExpr.R` (for the sensitivity and precision analysis run on the Bottomly et al. [2] dataset). The *Cuffdiff 2* commands are contained in the `inst/script/pickrell/` and `inst/script/bottomly/` directories.

Supplementary Tables

$i \in \{1, \ldots, n\}$	gene index
$j \in \{1, \ldots, m\}$	sample index
$r \in \{0, \ldots, p-1\}$	covariate index, with intercept $r = 0$
K_{ij}	counts of reads for gene i, sample j
μ_{ij}	fitted mean
α_i	gene-specific dispersion
s_j	sample-specific size factor
s_{ij}	gene- and sample-specific normalization factor
q_{ij}	proportional to true concentration of fragments
x_{jr}	elements of the design matrix X
β_{ir}	the logarithmic fold change for gene i and covariate r
$\bar{\mu}_i$	mean of normalized counts of gene i
σ_d^2	prior variance for logarithmic dispersions
σ_{lde}^2	sampling variance of logarithmic dispersion estimator
s_{lr}^2	variance estimate for logarithmic residuals of dispersion
α_i^{gw}	gene-wise dispersion estimate
$\alpha_{\text{tr}}(\bar{\mu}_i)$	trended dispersion fit
α_i^{MAP}	maximum *a posteriori* estimate of dispersion
σ_r^2	prior variance for logarithmic fold change r
Σ_i	covariance matrix for $\vec{\beta}_i$

Additional file 1: Table S1: Notation

m	p	α	theor. var.	sample var.
6	2	0.05	0.645	0.670
6	2	0.20	0.645	0.642
8	2	0.05	0.395	0.409
8	2	0.20	0.395	0.396
8	3	0.05	0.490	0.530
8	3	0.20	0.490	0.462
16	2	0.05	0.154	0.160
16	2	0.20	0.154	0.138
16	3	0.05	0.166	0.169
16	3	0.20	0.166	0.156

Additional file 1: Table S2: Theoretical and sample variance of logarithmic dispersion estimates for various combinations of sample size m, number of parameters p and true dispersion α. The estimates are the *DESeq2* gene-wise estimates from 4000 simulated genes with Negative Binomial counts with a mean of 1024. The sample variance of the logarithmic dispersion estimates is generally close to the approximation of theoretical variance.

function/package	version	additional information
DESeq (old)	1.16.0	using the GLM test
DESeq2	1.4.0	
edgeR	3.6.0	using GLM and trended dispersion estimation
DSS	2.2.0	
voom: *limma*	3.20.1	
SAMseq: *samr*	2.0	using `samr.pvalues.from.perms` for p-values
EBSeq	1.4.0	(1 − PPDE) used for FDR cutoff, following user guide
Cuffdiff 2	2.1.1	
GFOLD	1.1.2	
PoiClaClu	1.0.2	

Additional file 1: Table S3: Versions of software used in manuscript

Supplementary Figures

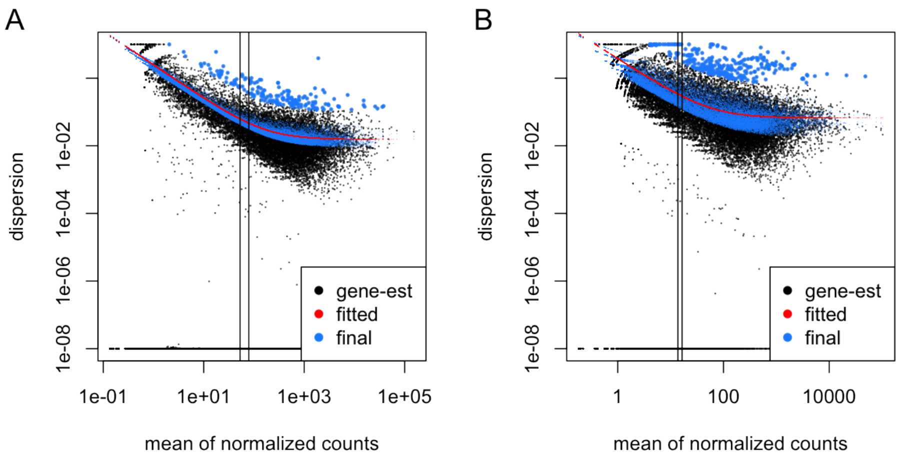

Additional file 1: Figure S1: **Shrinkage estimation of dispersion over all genes.** Plot of dispersion estimates over the average expression strength (A) for the Bottomly et al. [2] dataset with 6 samples across 2 conditions and (B) for the Pickrell et al. [1] dataset with 5 samples fitting only an intercept term. This plot shows the same data as Figure 1, but with dispersions drawn for all genes instead of only a subset. The points at the bottom of the plot typically arise from genes for which the observed variance is below the variance expected under a Poisson model. In such a case, the maximum-likelihood estimate will be essential zero, and appears here with the surrogate value 10^{-8}. Vertical lines indicate the reciprocal of the asymptotic dispersion α_0, on the scale of raw counts for the samples with the smallest and largest size factor. The lines hence mark the count range where Poisson noise and overdispersion contribute about equally to the observed variance. For very low count values (left of the lines), dispersion estimates become unreliable, causing possible overestimation (Methods).

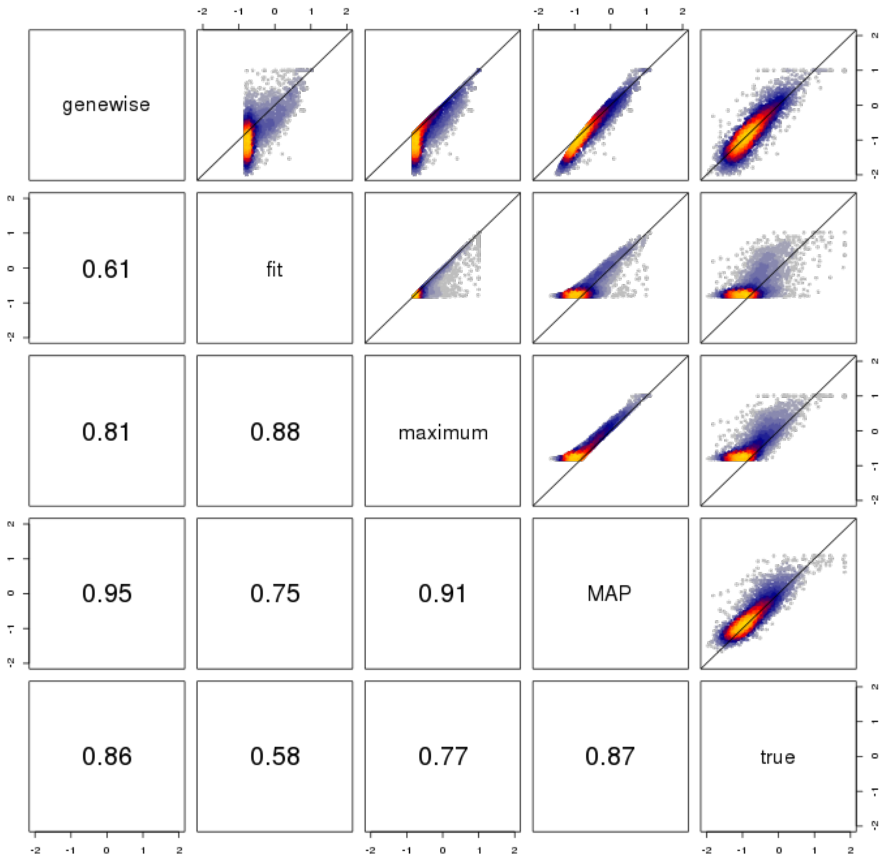

Additional file 1: Figure S2: **Scatterplot of various estimates of dispersion using *DESeq2*, against the true dispersion in the logarithmic scale (base 10) from simulated counts.** The blue, red, and yellow colors indicate regions of increasing density of points. Counts for 4000 genes and for 10 samples in two groups were simulated with no true difference in means. The Negative Binomial counts had mean and dispersion drawn from the joint distribution of the mean and gene-wise dispersion estimates from the Pickrell et al. dataset. The estimates shown are *genewise*, the CR-adjusted maximum likelihood estimate; *fit* the value from the fitted curve; *maximum*, the maximum of the two previous values (the estimate used in the older version of *DESeq*); and *MAP*, the maximum *a posteriori* estimate used in *DESeq2*. The correlations shown in the bottom panels do not include the very low gene-wise estimates of dispersion which can result in potential false positives. The *MAP*, shrunken estimates used in *DESeq2* were closer to the diagonal, while the *maximum* estimate was typically above the true value of dispersion, which can lead to overly-conservative inference of differential expression.

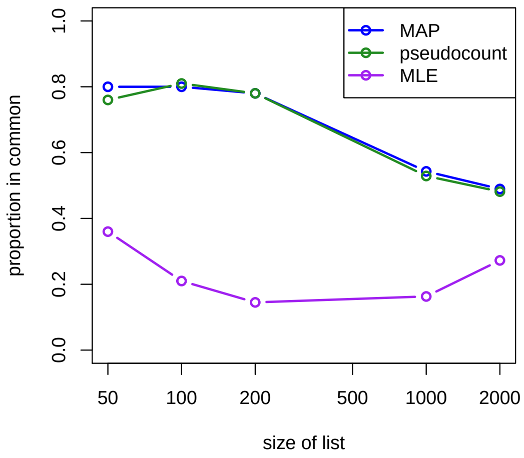

Additional file 1: Figure S3: **"Concordance at the top" plot.** *DESeq2* is run on equally split halves of the data of Bottomly et al. [2] and the proportion of genes in common after ranking by absolute logarithmic fold changes is compared [3]. On the y-axis is the number of genes in common between the splits divided by the size of the top-ranked list. The MAP estimate of logarithmic fold change and the MLE after adding a pseudocount of 1 to all samples provide nearly the same concordance for various cutoffs, while ranking by the MLE on raw counts has generally low concordance. For further demonstrations of the advantage of MAP over pseudocount, see section *Benchmarks*.

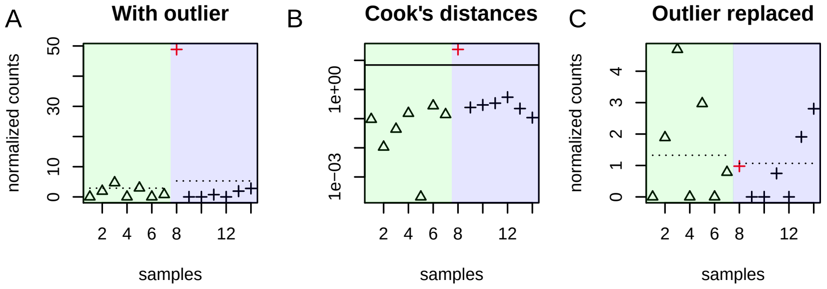

Additional file 1: Figure S4: **Cook's distance outlier detection**. Shown are normalized counts and Cook's distances for a 7 by 7 comparison of the Bottomly et al. [2] dataset. (A) Normalized counts for a single gene, samples divided into groups by strains (light green and light blue). Dotted segments represent fitted means. An apparent outlier is highlighted in red. (B) The Cook's distances for each sample for this gene, and the 99% quantile of the $F(p, m-p)$ cutoff used for flagging outliers. Note the logarithmic scaling of the y-axis. (C) The normalized counts after replacing the outlier with the trimmed mean over all samples, scaled by size factor. The fitted means now are less affected by the single outlier sample.

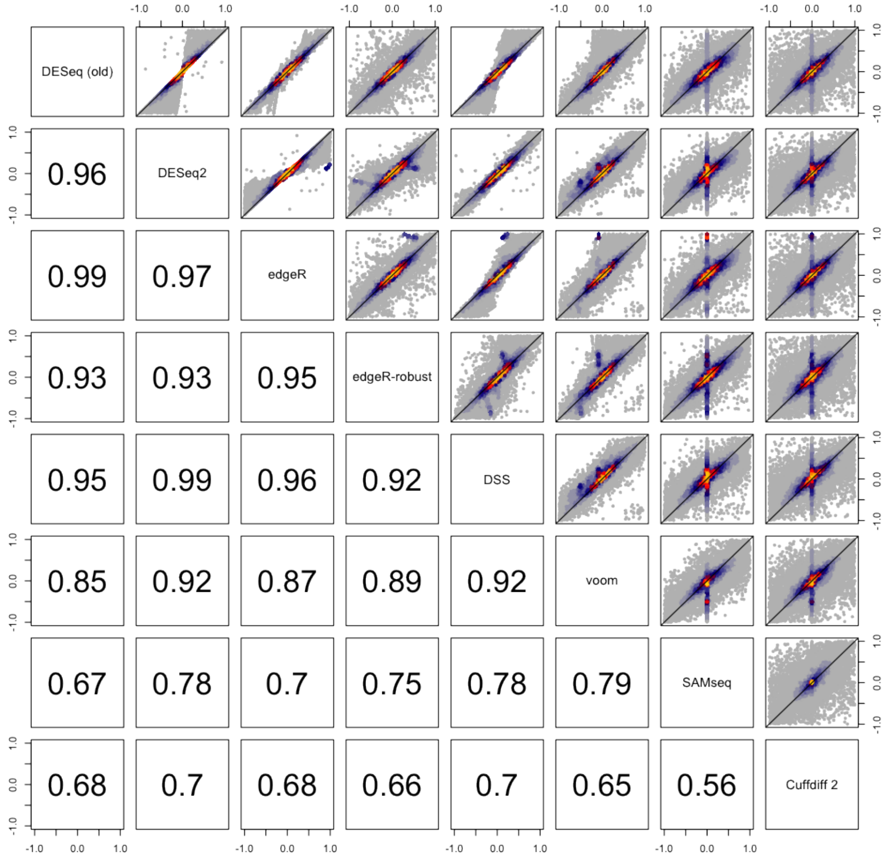

Additional file 1: Figure S5: **Scatterplots of estimated logarithmic fold changes from all algorithms.** $\log_2$ fold changes are estimated from one of the verification sets of the Bottomly et al. [2] dataset (see section *Benchmarks on RNA-seq data*). Bottom panels display the Pearson correlation coefficients. We note that the direction of the estimate of differential expression for *DESeq2* and *Cuffdiff 2* accorded for the majority of genes called differentially expressed: Among genes which were called differentially expressed by either of these two algorithms, both agreed on the sign of the estimated logarithmic fold change for 96% of genes (averaged over all 30 replicates) in the evaluation set and for 96% of genes in the verification set.

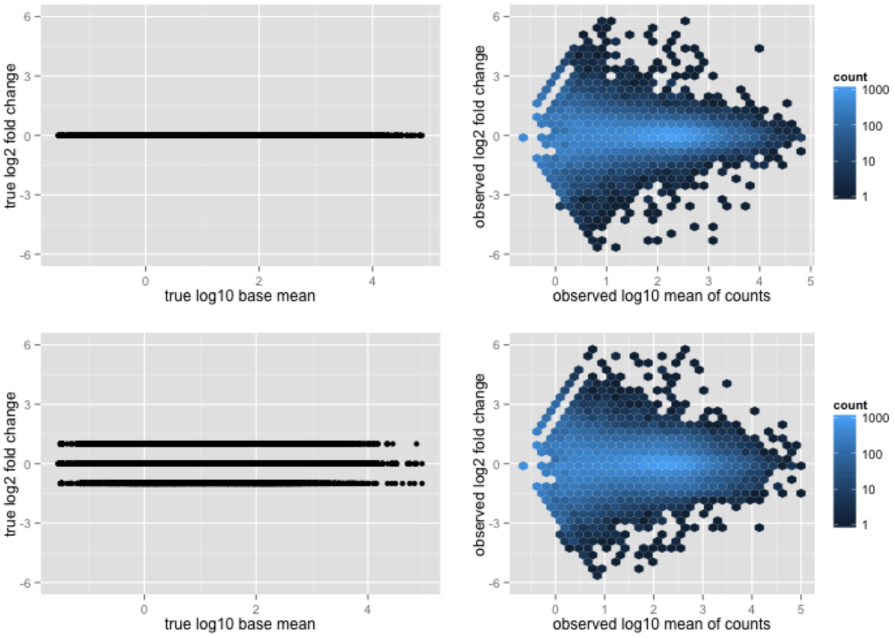

Additional file 1: Figure S6: **True logarithmic fold changes and the observed logarithmic fold changes induced by the simulation for differential expression.** The left plots show the true logarithmic fold changes and true logarithm of base mean, while the right plots show the observed logarithmic fold changes and observed logarithm of the mean of counts for a 4 vs 4 sample comparison. The observed logarithmic fold change was calculated as the logarithm of the mean of counts in one group divided by the mean of counts in the second group. In the top row, all true logarithmic fold changes were equal to zero. On the bottom row, 20% of true logarithmic fold changes were set to a fixed value as in the simulation benchmark for differential expression. We note that mean-independent fixed fold changes produced an MA-plot of observed logarithmic fold changes with mean dependence which is similar to that seen in real data, as in Additional file 1: Figure S7.

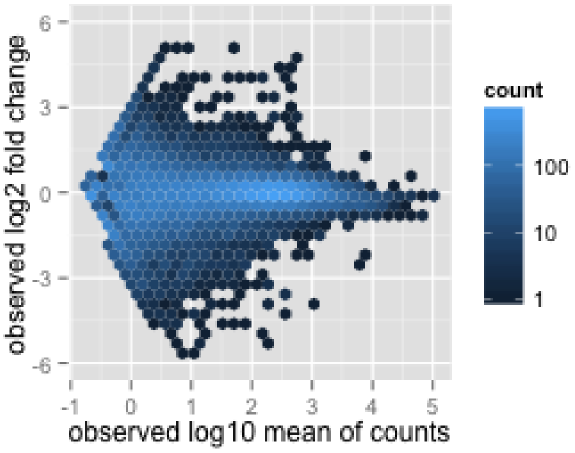

Additional file 1: Figure S7: **MA-plot from real data.** The observed logarithmic fold changes were generated from a 4 vs 4 sample comparison of the Pickrell et al. dataset, wherein there was no known phenotypic difference dividing the groups. The observed logarithmic fold change was calculated as the logarithm of the mean of normalized counts in one group divided by the mean of normalized counts in the second group. The observed mean of counts was calculated as the mean of normalized counts across all samples.

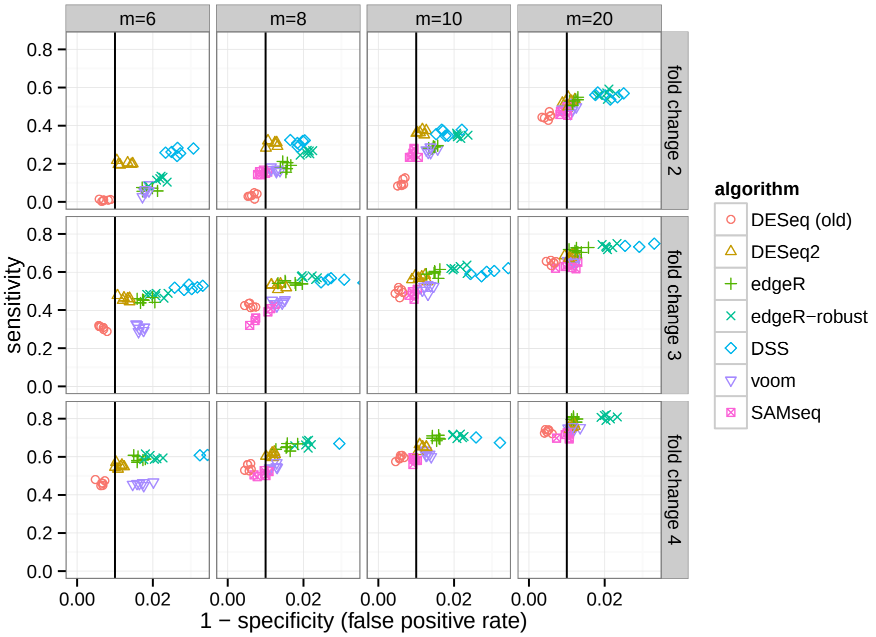

Additional file 1: Figure S8: **Use of simulation to assess the sensitivity and specificity of algorithms across combinations of sample size and effect size.** Shown are results for the benchmark through simulation described in the main text and in Figure 6. The sensitivity was calculated as the fraction of genes with adjusted p-value less than 0.1 among the genes with true differences between group means. The specificity was calculated as the fraction of genes with p-value greater than 0.01 among the genes with no true differences between group means. The p-value was chosen instead of the adjusted p-value, as this allows for comparison against the expected fraction of p-values less than a critical value given the uniformity of p-values under the null hypothesis. *DESeq2* often had the highest sensitivity of those algorithms which control the false positive rate, i.e., those algorithms which fall on or to the left of the vertical black line (1% p-values less than 0.01 for the non-DE genes). *EBSeq* results were not included in this plot as it returns posterior probabilities, which unlike p-values are not expected to be uniformly distributed under the null hypothesis.

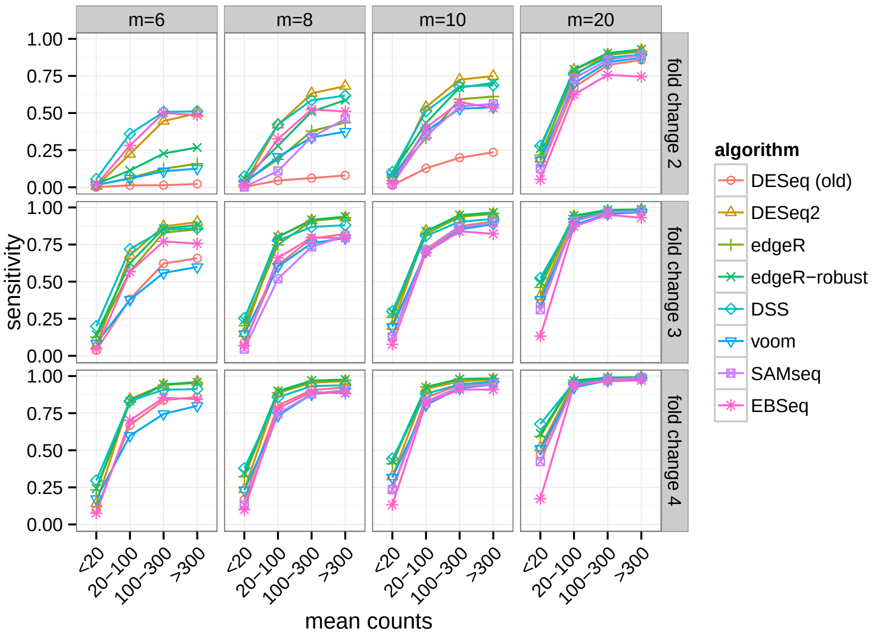

Additional file 1: Figure S9: **The dependence of sensitivity on the mean of counts for a gene in simulated data.** Shown are results for the benchmark through simulation described in Figure 6 and Additional file 1: Figure S8. The sensitivity of algorithms across combinations of sample size and effect size in the simulated datasets is further stratified by the mean of counts of the differentially expressed genes. The height of the sensitivity curves in this figure corresponds to those shown in Figure 6 and Additional file 1: Figure S8 which demonstrates the total sensitivity of each algorithm. Points indicate the average over 6 replicates. All algorithms show an expected dependence of sensitivity on the mean of counts. We note that *EBSeq* version 1.4.0 by default removes low count genes – whose 75% quantile of normalized counts is less than 10 – before differential expression calling.

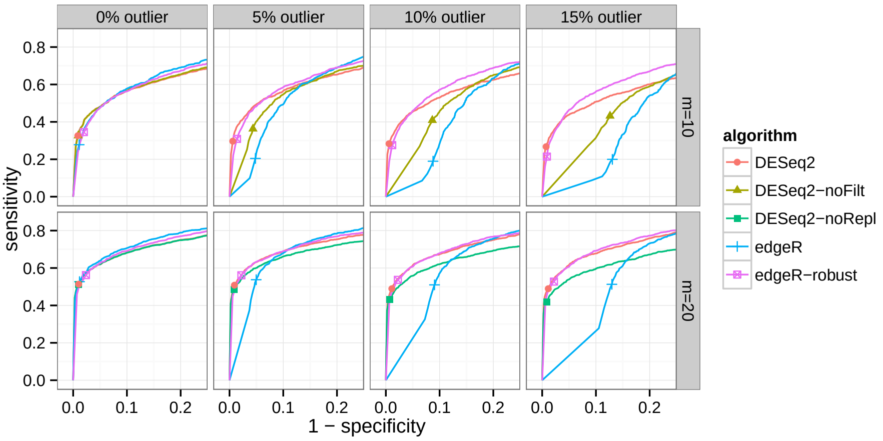

Additional file 1: Figure S10: **Sensitivity-specificity curves for detecting true differences in the presence of outliers.** Negative Binomial counts were simulated for 4000 genes and total sample sizes (m) of 10 and 20, for a two-group comparison. 80% of the simulated genes had no true differential expression, while for 20% of the genes true logarithmic (base 2) fold changes were randomly drawn from {-1, 1}. The number of genes with simulated outliers was increased from 0% to 15%. The outliers were constructed for a gene by multiplying the count of a single sample by 100. Sensitivity and specificity were calculated by thresholding on p-values. Points indicate an adjusted p-value cutoff of 0.1. *DESeq2* with the default settings and *edgeR* with the robust setting had higher area under the curve compared to running *edgeR* without the robust option, turning off *DESeq2* gene filtering, and turning off *DESeq2* outlier replacement. *DESeq2* filters genes with potential outliers for samples with 3 to 6 replicates and replaces outliers for samples with 7 or more replicates, hence the filtering can be turned off for the top row ($m = 10$) and the replacement can be turned off for the bottom row ($m = 20$).

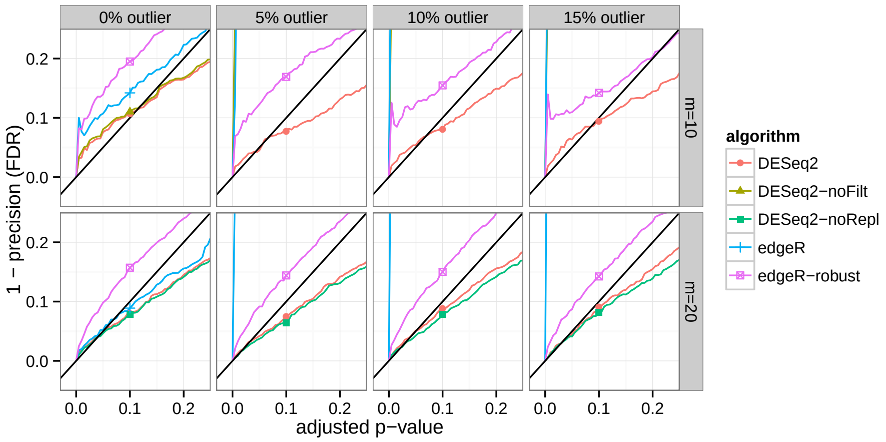

Additional file 1: Figure S11: **Outlier handling: One minus the precision (false discovery rate) plotted over various thresholds of adjusted *p*-value.** Shown are the results for the same simulation with outliers described in Additional file 1: Figure S10. Points indicate an adjusted *p*-value cutoff of 0.1. *edgeR* run with the robust setting had false discovery rate generally above the nominal value from the adjusted *p*-value threshold (black diagonal line). *DESeq2* run with default settings was generally at or below the line, which indicated control of the false discovery rate.

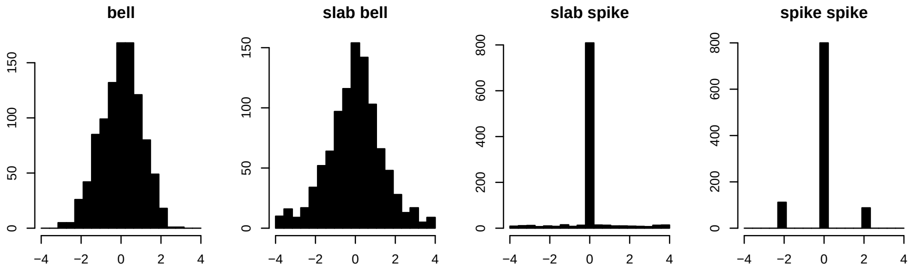

Additional file 1: Figure S12: **Benchmarking LFC estimation: Models for simulating logarithmic (base 2) fold changes.** For the *bell* model, true logarithmic fold changes were drawn from a Normal with mean 0 and variance 1. For the *slab bell* model, true logarithmic fold changes were drawn for 80% of genes from a Normal with mean 0 and variance 1 and for 20% of genes from a Uniform distribution with range from -4 to 4. For the *slab spike* model, true logarithmic fold changes were drawn similarly to the *slab bell* model except the Normal is replaced with a spike of logarithmic fold changes at 0. For the *spike spike* model, true logarithmic fold changes were drawn according to a spike of logarithmic fold changes at 0 (80%) and a spike randomly sampled from -2 or 2 (20%). These spikes represent fold changes of 1/4 and 4, respectively.

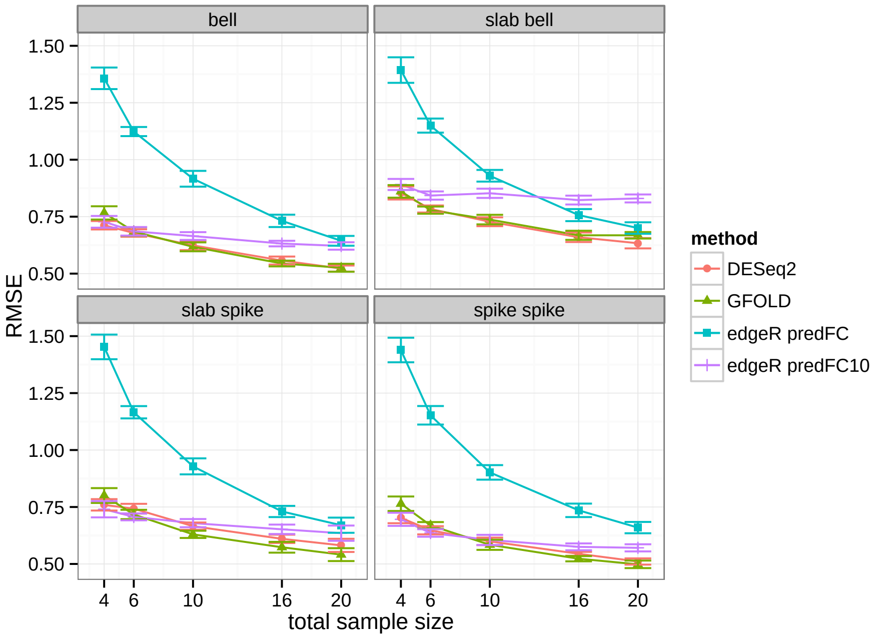

Additional file 1: Figure S13: **Root mean squared error (RMSE) for estimating logarithmic fold changes under the four models of logarithmic fold changes and varying total sample size m.** Simulated Negative Binomial counts were generated for two groups and for 1000 genes. Points and error bars are drawn for the mean and 95% confidence interval over 10 replicates. *DESeq2* and *GFOLD*, which both implement posterior logarithmic fold change estimates, had lower root mean squared error to the true logarithmic fold changes over all genes, compared to predictive logarithmic fold changes from edgeR, either using the default value of 0.125 for the *edgeR* argument *prior.count*, or after increasing *prior.count* to 10 (*edgeR* predFC10).

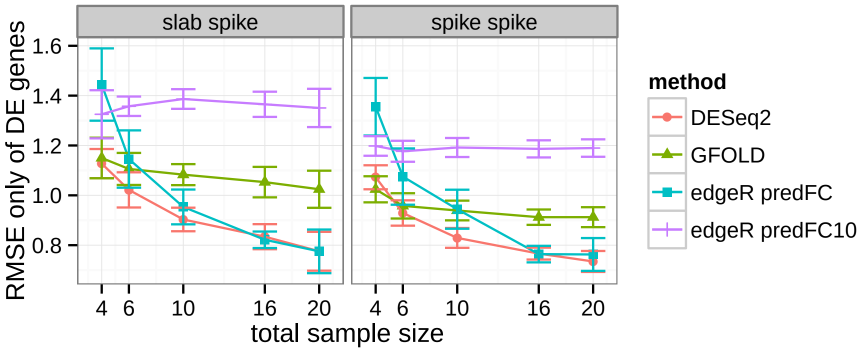

Additional file 1: Figure S14: **Root mean squared error (RMSE) of logarithmic fold change estimates, only considering genes with non-zero true logarithmic fold change.** For the same simulation as shown in Additional file 1: Figure S13, shown here is the error only for the 20% of genes with non-zero true logarithmic fold changes (for *bell* and *slab bell* all genes have non-zero logarithmic fold change). *DESeq2* had generally lower root mean squared error, compared to *GFOLD* which had higher error for large sample size and to *edgeR* which had higher error for low sample size.

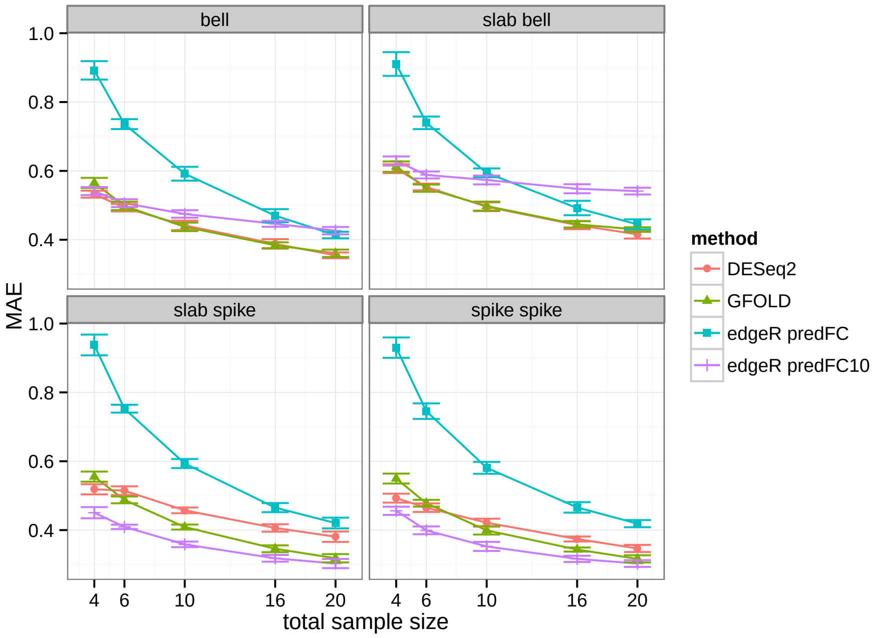

Additional file 1: Figure S15: **Mean absolute error (MAE) of logarithmic fold change estimates.** Results for the same simulation as shown in Additional file 1: Figure S13, however here using mean absolute error in place of root mean squared error. Mean absolute error places less weight on the largest errors. For the *bell* and *slab bell* models, *DESeq2* and *GFOLD* had the lowest mean absolute error, while for the *slab spike* and *spike spike* models, *GFOLD* and *edgeR* with a *prior.count* of 10 had lowest mean absolute error.

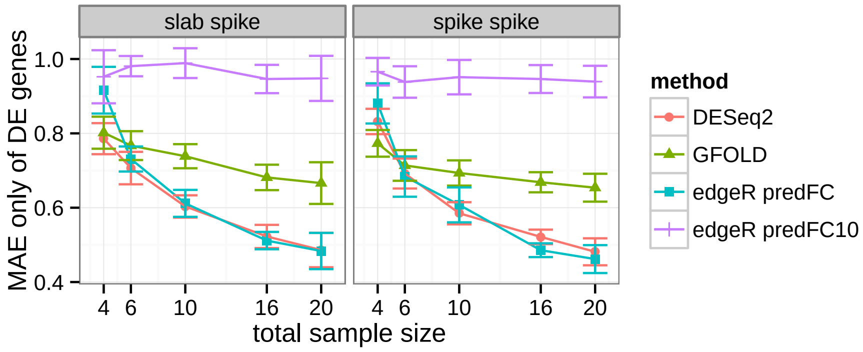

Additional file 1: Figure S16: **Mean absolute error (MAE) of logarithmic fold change estimates, only considering those genes with non-zero true logarithmic fold change.** While in Additional file 1: Figure S15, considering all genes for the *slab spike* and *spike spike* models, *GFOLD* and *edgeR* with a *prior.count* of 10 had lowest mean absolute error, the mean absolute error for these methods was relatively large for large sample size, when considering only the 20% of genes with true differentially expression. *DESeq2* and *edgeR* generally had the lowest mean absolute error.

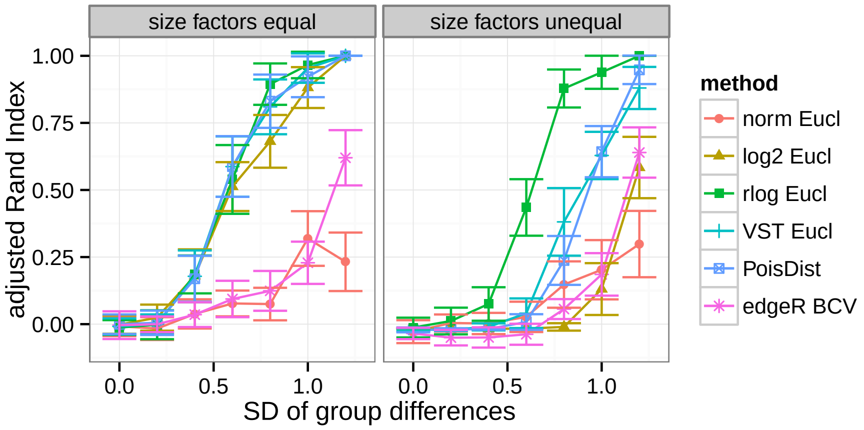

Additional file 1: Figure S17: **Adjusted Rand Index of clusters using various transformation and distances compared to the true clusters from simulation.** 4 simulated clusters with 4 samples each were generated using Negative Binomial counts over 2000 genes using the means and gene-wise estimates of dispersion from the Pickrell et al. dataset. 80% of genes were given equal mean across clusters, while for 20% of genes, logarithm (base 2) fold changes from a centroid were drawn from a zero-centered Normal distribution while varying the standard deviation (SD, x-axis). Larger standard deviation resulted in more distinct clusters, which are easier for the methods to recover. Simulation was performed with equal size factors, and with size factors for each group set to $[1, 1, \frac{1}{3}, 3]$. The methods assessed were: Euclidean distance on counts normalized by size factor, logarithm of normalized counts plus a pseudocount of 1, rlog transformed counts and variance stabilized counts (VST). Additionally, the Poisson Distance from the *PoiClaClu* package and the Biological Coefficient of Variation (BCV) distance from the *plotMDS* function of the *edgeR* package were used for hierarchical clustering. We note that the default distance used by *plotMDS* is not the BCV distance but more similar to the Euclidean distance of logarithmic counts. The points and error bars indicate the mean and 95% confidence interval from 20 replicates. In the simulations with equal size factors, the Poisson distance, the VST and the rlog had the highest accuracy in recovering true clusters. In the unequal size factor simulations, the rlog outperformed the other methods.

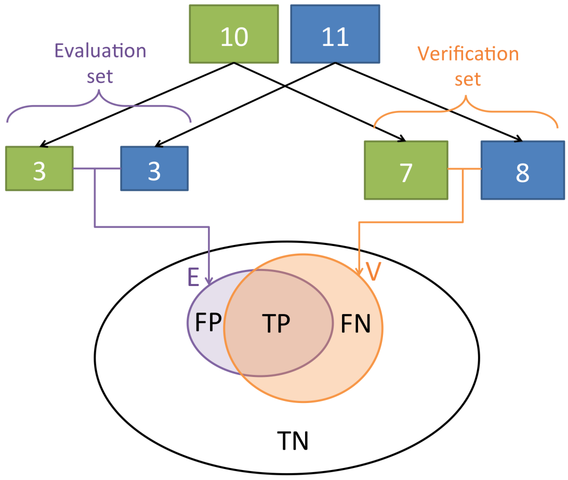

Additional file 1: Figure S18: **Diagram of the evaluation scheme for the benchmarks using real RNA-seq data.** The Bottomly et al. dataset with 10 and 11 replicates was split into a 3 vs 3 "evaluation set" and a 7 vs 8 "verification set". The positive calls from the verification set, denoted as set V, were taken as a pseudo-gold standard of truly differentially expressed genes. The algorithms were then evaluated based on the set E of positive calls in the evaluation set, comparing to the gold-standard calls from the set V. Sensitivity was calculated as $|E \cap V|/|V|$ and precision was calculated as $|E \cap V|/|E|$. Each algorithm's calls in the evaluation set were compared against each algorithm's calls in the verification set.

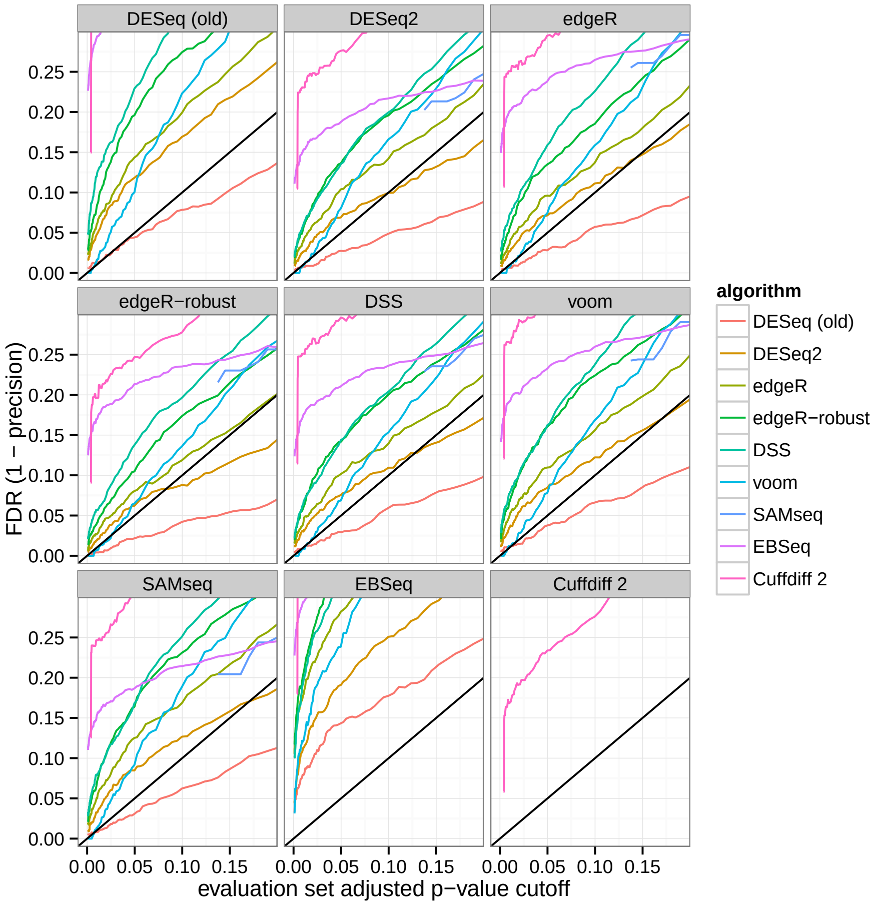

Additional file 1: Figure S19: **Actual versus nominal false discovery rate for the Bottomly et al. dataset.** The actual false discovery rate was calculated using the median of (1 − precision), though here varying the adjusted p-value cutoff, i. e., the nominal FDR, for the evaluation set (for *EBSeq*, the posterior probability of equal expression was used). A false positive was defined as a call in the evaluation set for a given critical value of adjusted p-value which did not have adjusted p-value less than 0.1 in the verification set. Ideally, curves should fall on the identity line (indicated by a black line); curves that fall above indicate that an algorithm is too permissive (anti-conservative), curves falling below indicate that an algorithm does not use its type-I error budget, i. e., is conservative. *DESeq2* had a false discovery rate nearly matching the nominal false discovery rate (black diagonal line) for the majority of algorithms used to determine the verification set calls. The old *DESeq* tool was often too conservative.

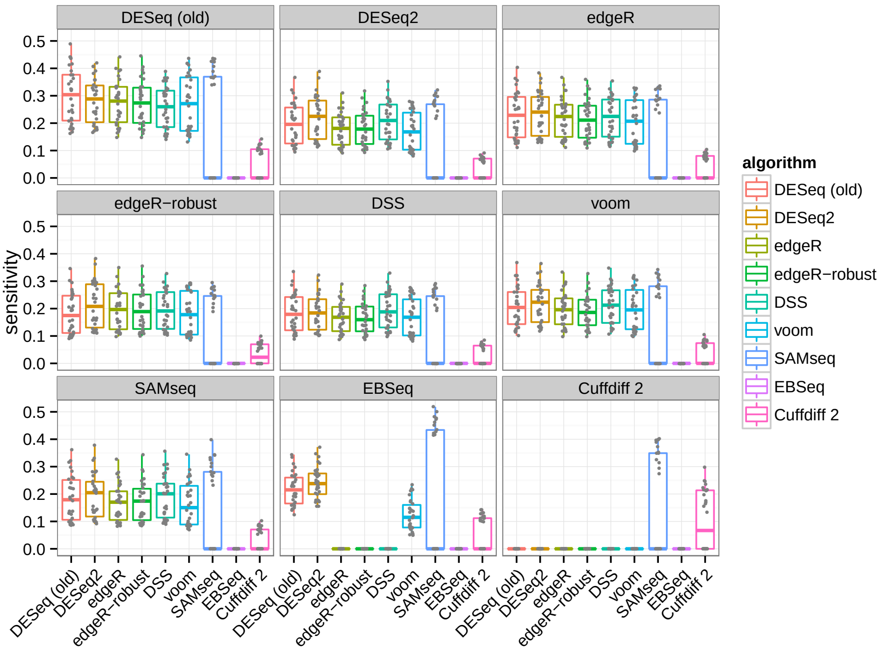

Additional file 1: Figure S20: **Sensitivity of algorithms evaluated while controlling the median precision.** While it was generally noted that sensitivity and precision were negatively correlated (Figures 8 and 9), here this effect was controlled by setting the adjusted p-value cutoff for the evaluation set calls such that the median precision of all algorithms would be 0.9 (actual false discovery rate of 0.1). This amounted to finding the point on the x-axis in Additional file 1: Figure S19, where the curve crosses 0.1 on the y-axis. For most algorithms, this meant setting an adjusted p-value cutoff below 0.1. *DESeq2* often had the highest median sensitivity for a given target precision, though the variability across random replicates was generally larger than the difference between algorithms.

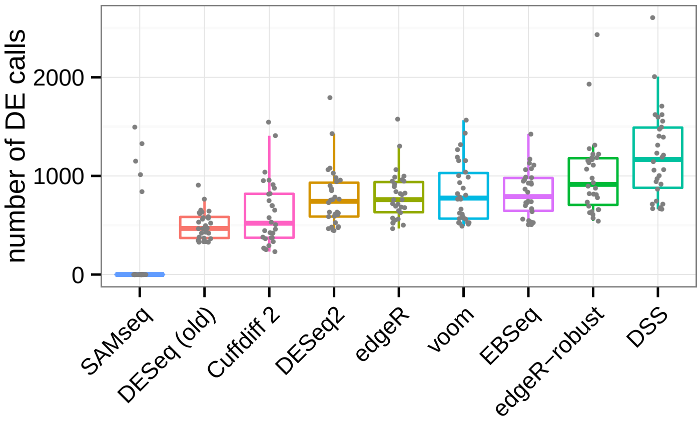

Additional file 1: Figure S21: **Number of total calls in the evaluation set (3 vs 3 samples)** of the sensitivity/precision analysis using the Bottomly et al. [2] dataset thresholding at adjusted p-value < 0.1, over 30 replications.

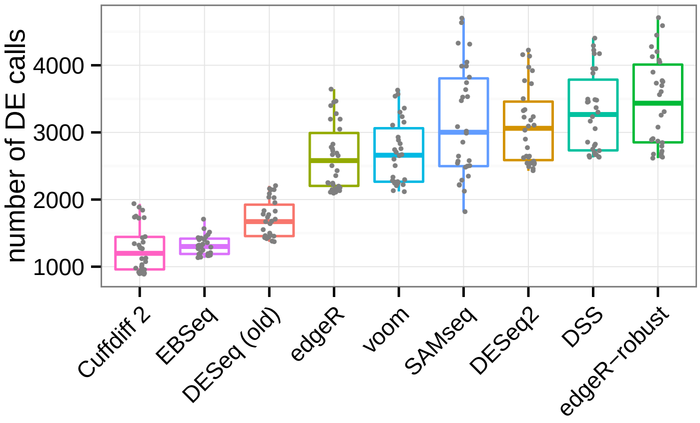

Additional file 1: Figure S22: **Number of total calls in the verification set (7 vs 8 samples)** of the sensitivity/precision analysis using the Bottomly et al. [2] dataset thresholding at adjusted p-value < 0.1, over 30 replications.

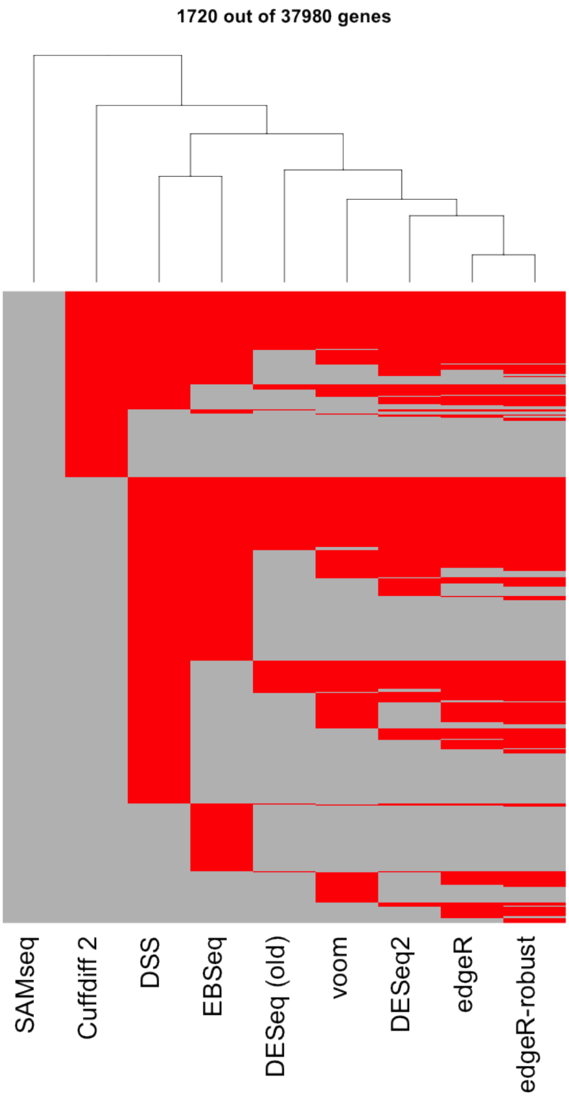

Additional file 1: Figure S23: **Clustering of each algorithm's calls on the evaluation set (3 vs 3 samples) for one replicate of the sensitivity/precision benchmark.** Genes are on the vertical axis and algorithms on the horizontal axis. Red lines indicate a gene had adjusted p-value < 0.1 in the evaluation set. Genes in which no algorithm had a call are not shown. Clustering is based on the Jaccard index.

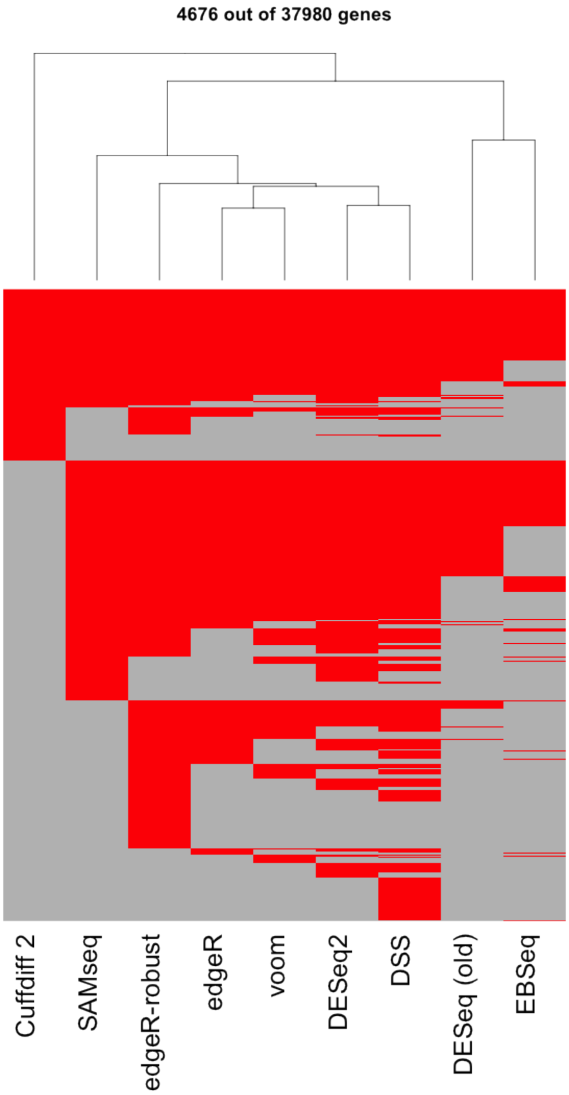

Additional file 1: Figure S24: **Clustering of algorithm calls on the verification set (7 vs 8 samples) for one replicate of the sensitivity/precision benchmark.** Genes are on the vertical axis and algorithms on the horizontal axis. Red lines indicate a gene had adjusted p-value < 0.1 in the verification set. Genes in which no algorithm had a call are not shown. Clustering is based on the Jaccard index.

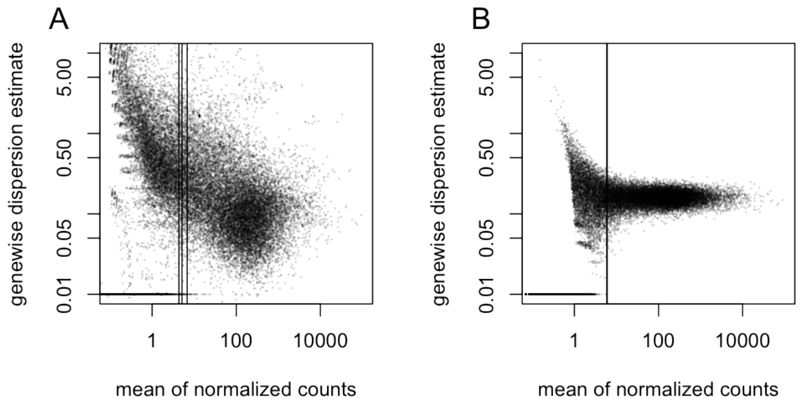

Additional file 1: Figure S25: **Demonstration through simulation that the dependence of dispersions on the mean seen in Figure 1B is not an artifact of estimation bias.** (A) The gene-wise estimates of dispersion for the 69 samples of the Pickrell et al. dataset. (B) The gene-wise estimates of dispersion for a simulated Negative Binomial dataset, using a fixed dispersion of $\alpha = 0.16$, equal to the asymptotic gene-wise dispersion estimate α_0 seen in the original dataset (A), and with the same means and the same number of genes and samples as the original dataset. Genes with dispersion estimates below the plotting range are depicted at the bottom of the frame. For genes with mean counts greater than ~ 5, the gene-wise dispersion estimates do not exhibit a dependence on the mean count for the simulated data in panel B. Vertical lines indicate the reciprocal of the asymptotic dispersion α_0, on the scale of raw counts for the 1st, 2nd and 3rd quartile of the size factors.

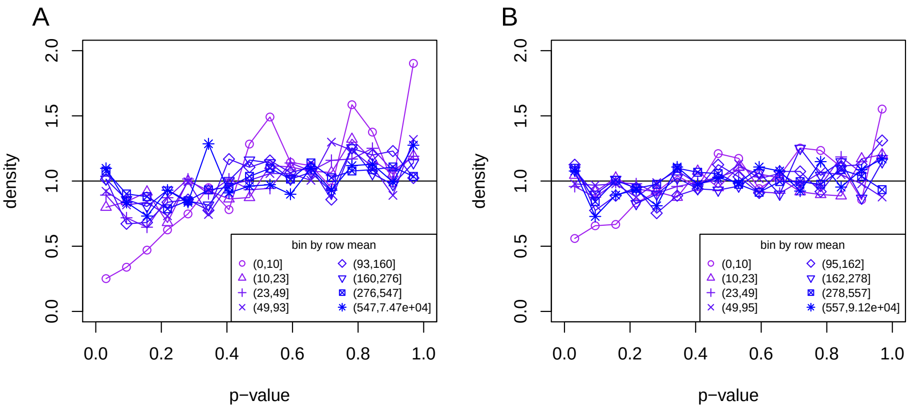

Additional file 1: Figure S26: **Marginal null histogram of the test statistic, p-values, conditioning on the filter statistic, the row mean of normalized counts across all samples, used for independent filtering.** A simulated dataset was constructed with (A) 6 samples or (B) 12 samples and 20,000 genes. In either case the samples were equally divided into 2 groups with no true difference between the means of the two groups. The means and dispersions of the Negative Binomial simulated data were drawn from the estimates from the Pickrell et al. dataset, and the standard *DESeq2* pipeline was run. The histogram of p-values was estimated at 16 equally spaced intervals spanning $[0, 1]$. The marginal distributions of the test statistic were generally uniform while conditioning on bins based on the filter statistic. The row mean bin with the smallest mean of normalized counts (mean count $0 - 10$) was depleted of small p-values. The black line indicates the expected frequency for a Uniform distribution.

References

[1] Pickrell, J.K., Marioni, J.C., Pai, A.A., Degner, J.F., Engelhardt, B.E., Nkadori, E., Veyrieras, J.-B., Stephens, M., Gilad, Y., Pritchard, J.K.: Understanding mechanisms underlying human gene expression variation with RNA sequencing. Nature **464**(7289), 768–772 (2010)

[2] Bottomly, D., Walter, N.A.R., Hunter, J.E., Darakjian, P., Kawane, S., Buck, K.J., Searles, R.P., Mooney, M., McWeeney, S.K., Hitzemann, R.: Evaluating gene expression in C57BL/6J and DBA/2J mouse striatum using RNA-seq and microarrays. PLoS ONE **6**(3), 17820 (2011)

[3] Irizarry, R.A., Warren, D., Spencer, F., Kim, I.F., Biswal, S., Frank, B.C., Gabrielson, E., Garcia, J.G.N., Geoghegan, J., Germino, G., Griffin, C., Hilmer, S.C., Hoffman, E., Jedlicka, A.E., Kawasaki, E., Martínez-Murillo, F., Morsberger, L., Lee, H., Petersen, D., Quackenbush, J., Scott, A., Wilson, M., Yang, Y., Ye, S.Q., Yu, W.: Multiple-laboratory comparison of microarray platforms. Nat Methods **2**(5), 345–350 (2005)
